# Nanotoxicology: An Emerging Discipline Evolving from Studies of Ultrafine Particles

**DOI:** 10.1289/ehp.7339

**Published:** 2005-03-22

**Authors:** Günter Oberdörster, Eva Oberdörster, Jan Oberdörster

**Affiliations:** ^1^Department of Environmental Medicine, University of Rochester, Rochester, New York, USA; ^2^Department of Biology, Southern Methodist University, Dallas, Texas, USA; ^3^Toxicology Department, Bayer CropScience, Research Triangle Park, North Carolina, USA

**Keywords:** biokinetics, central nervous system, engineered nanomaterials, environmental health, human health, nanosized particles, respiratory tract, risk assessment, skin, ultrafine particles

## Abstract

Although humans have been exposed to airborne nanosized particles (NSPs; < 100 nm) throughout their evolutionary stages, such exposure has increased dramatically over the last century due to anthropogenic sources. The rapidly developing field of nanotechnology is likely to become yet another source through inhalation, ingestion, skin uptake, and injection of engineered nanomaterials. Information about safety and potential hazards is urgently needed. Results of older bio-kinetic studies with NSPs and newer epidemiologic and toxicologic studies with airborne ultrafine particles can be viewed as the basis for the expanding field of nanotoxicology, which can be defined as safety evaluation of engineered nanostructures and nanodevices. Collectively, some emerging concepts of nanotoxicology can be identified from the results of these studies. When inhaled, specific sizes of NSPs are efficiently deposited by diffusional mechanisms in all regions of the respiratory tract. The small size facilitates uptake into cells and transcytosis across epithelial and endothelial cells into the blood and lymph circulation to reach potentially sensitive target sites such as bone marrow, lymph nodes, spleen, and heart. Access to the central nervous system and ganglia via translocation along axons and dendrites of neurons has also been observed. NSPs penetrating the skin distribute via uptake into lymphatic channels. Endocytosis and biokinetics are largely dependent on NSP surface chemistry (coating) and *in vivo* surface modifications. The greater surface area per mass compared with larger-sized particles of the same chemistry renders NSPs more active biologically. This activity includes a potential for inflammatory and pro-oxidant, but also antioxidant, activity, which can explain early findings showing mixed results in terms of toxicity of NSPs to environmentally relevant species. Evidence of mitochondrial distribution and oxidative stress response after NSP endocytosis points to a need for basic research on their interactions with subcellular structures. Additional considerations for assessing safety of engineered NSPs include careful selections of appropriate and relevant doses/concentrations, the likelihood of increased effects in a compromised organism, and also the benefits of possible desirable effects. An interdisciplinary team approach (e.g., toxicology, materials science, medicine, molecular biology, and bioinformatics, to name a few) is mandatory for nanotoxicology research to arrive at an appropriate risk assessment.

Exposures to airborne nanosized particles (NSPs; < 100 nm) have been experienced by humans throughout their evolutionary stages, but it is only with the advent of the industrial revolution that such exposures have increased dramatically because of anthropogenic sources such as internal combustion engines, power plants, and many other sources of thermo-degradation. The rapidly developing field of nanotechnology is likely to become yet another source for human exposures to NSPs—engineered nanoparticles (NPs)—by different routes: inhalation (respiratory tract), ingestion [gastrointestinal (GI) tract], dermal (skin), and injection (blood circulation). Table 1 summarizes some of the natural and anthropogenic sources of NSPs, the latter divided into unintentional and intentional sources.

Biologically based or naturally occurring molecules that are found inside organisms since the beginning of life can serve as model nanosized materials. For example, biogenic magnetite is a naturally occurring NSP that occurs in many species ranging from bacteria to protozoa to animals (Blakemore 1975; Kirschvink et al. 2001). Biogenic magnetite has even been found in brains of humans (Dunn et al. 1995; Kirschvink et al. 1992; Schultheiss-Grassi et al. 1999) and has been associated with neurodegenerative diseases (Dobson 2001; Hautot et al. 2003). A biologic model of coated nanomaterials can be found in ferritin, which is an approximately 12-nm-large iron storage protein that contains 5- to 7-nm-sized hydrous ferric oxide phosphate inside a protective protein shell (Donlin et al. 1998). Nanosized materials, including fullerenes, occur naturally from combustion processes such as forest fires and volcanoes.

Obvious differences between unintentional and intentional anthropogenic NSPs are the polydispersed and chemically complex nature (elemental, soluble, and volatile carbon compounds; soluble and poorly soluble inorganics; Cyrys et al. 2003; Hughes et al. 1998) of the former, in contrast to the monodisperse and precise chemically engineered characteristics and solid form of the latter, generated in gas or liquid phase [National Nanotechnology Initiative (NNI) 2004]. However, despite these differences, the same toxicologic principles are likely to apply for NPs, because not only size but also a number of other particle parameters determine their biologic activity. The multitude of interactions of these factors has yet to be assessed, and in this article we attempt to summarize our present knowledge.

NSPs are variably called ultrafine particles (UFPs) by toxicologists [U.S. Environmental Protection Agency (EPA) 2004], Aitken mode and nucleation mode particles by atmospheric scientists [Kulmala 2004; National Research Council (NRC) 1983], and engineered nanostructured materials by materials scientists (NNI 2004). Figure 1 depicts the range of sizes of airborne ambient particulate matter, including the nucleation-mode, Aitken-mode, accumulation-mode, and coarse-mode particles. Ambient particles < 0.1 μm, defined as UFPs in the toxicologic literature, consist of transient nuclei or Aitken nuclei (NRC 1983). More recently, even smaller particles in the nucleation mode with peak diameters around 4 nm have been observed (McMurry and Woo 2002). The distinction between NSPs generated by internal combustion engines and NPs becomes further clouded by the finding of nanotubes in diesel exhaust (Evelyn et al. 2003). The use of the term “nano” in this review reflects only particle size and not chemical composition. For the purposes of this review, we use the following terms: “Nanosized particle” (NSP) includes all engineered and ambient nanosized spherical particles < 100 nm. “Engineered nanoparticle” (NP) includes only spherical NSPs specifically engineered in the laboratory; other engineered nanosized structures will be labeled according to their shape, for example, nanotubes, nanofibers, nanowires, nanorings, and so on. “Ultrafine particle” (UFP) includes ambient and laboratory-generated NSPs that are not produced in a controlled, engineered way.

Table 2 shows the tremendous differences in particle number concentrations and particle surface areas for particles of the four ambient modes, assuming an airborne concentration of 10 μg/m^3^ of unit density particles of each size. The extraordinarily high number concentrations of NSPs per given mass will likely be of toxicologic significance when these particles interact with cells and subcellular components. Likewise, their increased surface area per unit mass can be toxicologically important if other characteristics such as surface chemistry and bulk chemistry are the same. Although the mass of UFPs in ambient air is very low, approaching only 0.5–2 μg/m^3^ at background levels (Hughes et al. 1998), it can increase several-fold during high pollution episodes or on highways (Brand et al. 1991; Shi et al. 2001; Zhu et al. 2002).

## 

### Physicochemical characteristics as determinants of biologic activity.

The small size and corresponding large specific surface area of solid NSPs (Table 2) confer specific properties to them, for example, making them desirable as catalysts for chemical reactions. The importance of surface area becomes evident when considering that surface atoms or molecules play a dominant role in determining bulk properties (Amato 1989); the ratio of surface to total atoms or molecules increases exponentially with decreasing particle size (Figure 2). Increased surface reactivity predicts that NSPs exhibit greater biologic activity per given mass compared with larger particles, should they be taken up into living organisms and provided they are solid rather than solute particles. This increased biologic activity can be either positive and desirable (e.g., antioxidant activity, carrier capacity for therapeutics, penetration of cellular barriers for drug delivery) or negative and undesirable (e.g., toxicity, induction of oxidative stress or of cellular dysfunction), or a mix of both. Not only may adverse effects be induced, but interactions of NSPs with cells and subcellular structures and their biokinetics are likely to be very different from those of larger-sized particles. For example, more than 60 years ago virologists described the translocation of 30- to 50-nm-sized virus particles along axons and dendrites of neurons and across epithelia (Howe and Bodian 1940), whereas first reports about increased inflammatory activity and epithelial translocation of man-made 20- and 30-nm solid particles appeared only more recently (Ferin et al. 1990; Oberdörster et al. 1990).

The characteristic biokinetic behaviors of NPs are attractive qualities for promising applications in medicine as diagnostic and therapeutic devices and as tools to investigate and understand molecular processes and structures in living cells (Akerman et al. 2002; Foley et al. 2002; Kreuter 2001; Li et al. 2003). For example, targeted drug delivery to tissues that are difficult to reach [e.g., central nervous system (CNS)], NPs for the fight against cancer, intra-vascular nanosensor and nanorobotic devices, and diagnostic and imaging procedures are presently under development. The discipline of nanomedicine—defined as medical application of nanotechnology and related research—has arisen to design, test, and optimize these applications so that they can eventually be used routinely by physicians (Freitas 1999).

However, in apparent stark contrast to the many efforts aimed at exploiting desirable properties of NPs for improving human health are the limited attempts to evaluate potential undesirable effects of NPs when administered intentionally for medicinal purposes, or after unintentional exposure during manufacture or processing for industrial applications. The same properties that make NPs so attractive for development in nanomedicine and for specific industrial processes could also prove deleterious when NPs interact with cells. Thus, evaluating the safety of NPs should be of highest priority given their expected worldwide distribution for industrial applications and the likelihood of human exposure, directly or through release into the environment (air, water, soil). Nanotoxicology—an emerging discipline that can be defined as “science of engineered nanodevices and nanostructures that deals with their effects in living organisms”—is gaining increased attention. Nanotoxicology research not only will provide data for safety evaluation of engineered nanostructures and devices but also will help to advance the field of nanomedicine by providing information about their undesirable properties and means to avoid them.

### Human exposure to nanosized materials.

In addition to natural and anthropogenic sources of UFPs in the ambient air, certain workplace conditions also generate NSPs that can reach much higher exposure concentrations, up to several hundred micrograms per cubic meter, than is typically found at ambient levels. In contrast to airborne UFP exposures occurring via inhalation at the workplace and from ambient air, not much is known about levels of exposure via different routes for NPs, whether it is by direct human exposure or indirectly through contamination of the environment. For example, are there or will there be significant exposures to airborne singlet engineered carbon nanotubes or C_60_ fullerenes? First measurements at a model workplace gave only very low concentrations, < 50 μg/m^3^, and these were most likely in the form of aggregates (Maynard et al. 2004). However, even very low concentrations of nanosized materials in the air represent very high particle number concentrations, as is well known from measurements of ambient UFPs (Hughes et al. 1998). For example, a low concentration of 10 μg/m^3^ of unit density 20-nm particles translates into > 1 × 10^6^ particles/cm^3^ (Table 2). Inhalation may be the major route of exposure for NPs, yet ingestion and dermal exposures also need to be considered during manufacture, use, and disposal of engineered nanomaterials, and specific biomedical applications for diagnostic and therapeutic purposes will require intravenous, subcutaneous, or intramuscular administration (Table l). It can be assumed, however, that the toxicology of NPs can build on the experience and data already present in the toxicology literature of ambient UFPs. [Additional details provided in Supplemental Material available online (http://ehp.niehs.nih.gov/members/2005/7339/supplemental.pdf).]

### Manufactured nanomaterials in the environment.

Manufactured nanomaterials are likely to enter the environment for several reasons. Some are and others will be produced by the ton, and some of any material produced in such mass quantities is likely to reach the environment from manufacturing effluent or from spillage during shipping and handling. They are being used in personal-care products such as cosmetics and sunscreens and can therefore enter the environment on a continual basis from washing off of consumer products (Daughton and Ternes 1999). They are being used in electronics, tires, fuel cells, and many other products, and it is unknown whether some of these materials may leak out or be worn off over the period of use. They are also being used in disposable materials such as filters and electronics and may therefore reach the environment through landfills and other methods of disposal.

Scientists have also found ways of using nanomaterials in remediation. Although many of these are still in testing stages (Chitose et al. 2003; Jaques and Kim 2000; Joo et al. 2004; Nagaveni et al. 2004; Nghiem et al. 2004; Tungittiplakorn et al. 2004), dozens of sites have already been injected with various nanomaterials, including nano-iron (Mach 2004). Testing to determine the safety of these NPs used for remediation to environmentally relevant species has not yet been done. Although most people are concerned with effects on large wildlife, the basis of many food chains depends on the benthic and soil flora and fauna, which could be dramatically affected by such NP injections. In addition, as noted by Lecoanet et al. (2004), nanosized materials may not migrate through soils at rapid enough rates to be valuable in remediation. Future laboratory and field trials will help clarify the line between remediation and contamination [Supplemental Material available online (http://ehp.niehs.nih.gov/members/2005/7339/supplemental.pdf)].

## Toxicology of Airborne UFPs

In recent years, interest in potential effects of exposure to airborne UFPs has increased considerably, and studies have shown that they can contribute to adverse health effects in the respiratory tract as well as in extrapulmonary organs. Results on direct effects of ambient and model UFPs have been reported from epidemiologic studies and controlled clinical studies in humans, inhalation/instillation studies in rodents, or *in vitro* cell culture systems. For example, several epidemiologic studies have found associations of ambient UFPs with adverse respiratory and cardiovascular effects resulting in morbidity and mortality in susceptible parts of the population (Pekkanen et al. 1997; Penttinen et al. 2001; Peters et al. 1997a, 1997b; von Klot et al. 2002; Wichmann et al. 2002), whereas other epidemiologic studies have not seen such associations (Pekkanen et al. 1997; Tiittanen et al. 1999). Controlled clinical studies evaluated deposition and effects of laboratory-generated UFPs. High deposition efficiencies in the total respiratory tract of healthy subjects were found, and deposition was even greater in subjects with asthma or chronic obstructive pulmonary disease. In addition, effects on the cardiovascular system, including blood markers of coagulation and systemic inflammation and pulmonary diffusion capacity, were observed after controlled exposures to carbonaceous UFPs (Anderson et al. 1990; Brown et al. 2002; Chalupa et al. 2004; Henneberger et al., 2005; Jaques and Kim 2000; Pekkanen et al. 2002; Pietropaoli et al. 2004; Wichmann et al. 2000).

Studies in animals using laboratory-generated model UFPs or ambient UFPs showed that UFPs consistently induced mild yet significant pulmonary inflammatory responses as well as effects in extrapulmonary organs. Animal inhalation studies included the use of different susceptibility models in rodents, with analysis of lung lavage parameters and lung histopathology, effects on the blood coagulation cascade, and translocation studies to extra-pulmonary tissues (Elder et al. 2000, 2002, 2004; Ferin et al. 1991; Ferin and Oberdörster 1992; Kreyling et al. 2002; Li et al. 1999; Nemmar et al. 1999, 2002a, 2002b, 2003; Oberdörster et al. 1992a, 1995, 2000, 2002, 2004; Semmler et al. 2004; Zhou et al. 2003).

**Figure 3:**
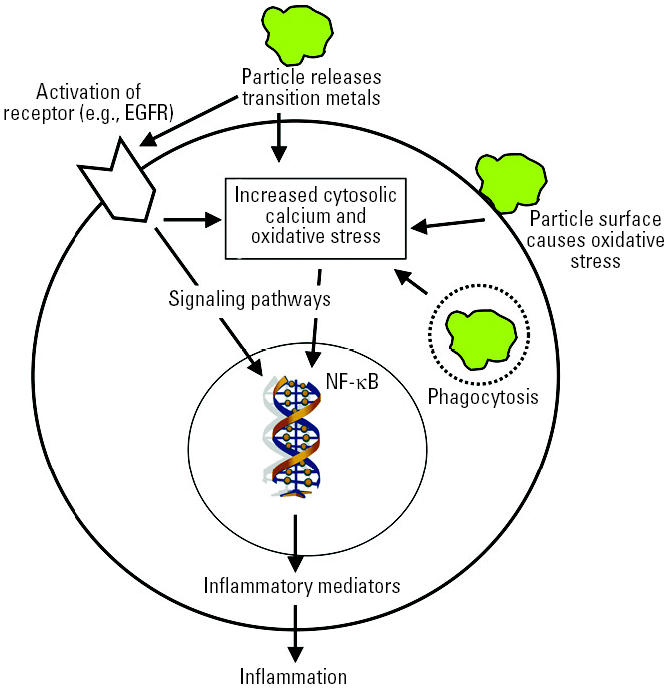
Hypothetical cellular interaction of NSPs (adapted from Donaldson and Tran 2002). EGFR, epidermal growth factor receptor. Inflammation and oxidative stress can be mediated by several primary pathways: *a*) the particle surface causes oxidative stress resulting in increased intracellular calcium and gene activation; *b*) transition metals released from particles result in oxidative stress, increased intracellular calcium, and gene activation; *c*) cell surface receptors are activated by transition metals released from particles, resulting in subsequent gene activation; or *d*) intracellular distribution of NSPs to mitochondria generates oxidative stress.

*In vitro* studies using different cell systems showed varying degrees of proinflammatory-and oxidative-stress–related cellular responses after dosing with laboratory-generated or filter-collected ambient UFPs (Brown et al. 2000, 2001; Li et al. 2003). Collectively, the *in vitro* results have identified oxidative-stress–related changes of gene expression and cell signaling pathways as underlying mechanisms of UFP effects, as well as a role of transition metals and certain organic compounds on combustion-generated UFPs (Figure 3). These can alter cell signaling pathways, including Ca^2+^ signaling and cytokine signaling (e.g., interleukin-8) (Donaldson et al. 2002; Donaldson and Stone 2003). Effects were on a mass basis greater for model UFPs than for fine particles, whereas effects for ambient UFP cellular responses were sometimes greater and sometimes less than those of fine and coarse particles. The interpretation of the *in vitro* studies is oftentimes difficult because particles of different chemical compositions were used, target cells were different, and duration, end points, and generally high dose levels also differed. Results from high doses in particular should be viewed with caution if they are orders of magnitude higher than predicted from relevant ambient exposures (see “Exposure dose–response considerations,” below). [Supplemental Material available online (http://ehp.niehs.nih.gov/members/2005/7339/supplemental.pdf).]

## Concepts of Nanotoxicology

### 

#### Laboratory rodent studies.

With respect to potential health effects of NSPs, two examples should serve to illustrate *a*) that NSPs have a higher inflammatory potential per given mass than do larger particles, provided they are chemically the same, and *b*) that NSPs generated under certain occupational conditions can elicit severe acute lung injury.

**Figure 4:**
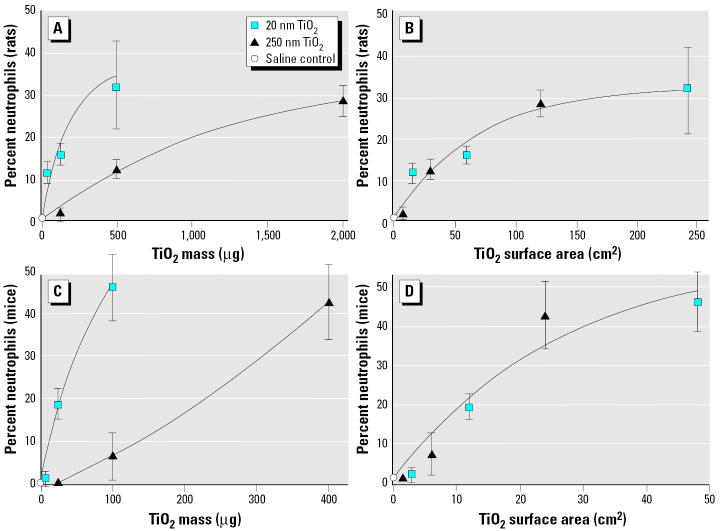
Percentage of neutrophils in lung lavage of rats (*A,B*) and mice (*C,D*) as indicators of inflammation 24 hr after intratracheal instillation of different mass doses of 20-nm and 250-nm TiO_2_ particles in rats and mice. (*A,C*) The steeper dose response of nanosized TiO_2_ is obvious when the dose is expressed as mass. (*B,D*) The same dose response relationship as in (*A,C*) but with dose expressed as particle surface area; this indicates that particle surface area seems to be a more appropriate dosemetric for comparing effects of different-sized particles, provided they are of the same chemical structure (anatase TiO_2_ in this case). Data show mean ± SD.

The first example involves studies with ultrafine and fine titanium dioxide (TiO_2_) particles, which showed that ultrafine anatase TiO_2_ (20 nm), when instilled intratracheally into rats and mice, induced a much greater pulmonary-inflammatory neutrophil response (determined by lung lavage 24 hr after dosing) than did fine anatase TiO_2_ (250 nm) when both types of particles were instilled at the same mass dose (Figure 4A,C). However, when the instilled dose was expressed as particle surface area, it became obvious that the neutrophil response in the lung for both ultrafine and fine TiO_2_ fitted the same dose–response curve (Figure 4B,D), suggesting that particle surface area for particles of different sizes but of the same chemistry, such as TiO_2_, is a better dosemetric than is particle mass or particle number (Oberdörster G 2000). Moreover, normalizing the particle surface dose to lung weight shows excellent agreement of the inflammatory response in both rats and mice [Figure S-2 in Supplemental Material available online (http://ehp.niehs.nih.gov/members/2005/7339/supplemental.pdf)]. The better fit of dose–response relationships by expressing the dose as surface area rather than mass when describing toxicologic effects of inhaled solid particles of different sizes has been pointed out repeatedly, especially when particles of different size ranges—nano to fine—were studied (Brown et al. 2001; Donaldson et al. 1998, 2002; Driscoll 1996; Oberdörster and Yu 1990; Oberdörster et al. 1992a; Tran et al. 1998, 2000) [Supplemental Material available online (http://ehp.niehs.nih.gov/members/2005/7339/supplemental.pdf)].

Particle chemistry, and specifically surface chemistry, plays a decisive role in addition to particle size, as shown in the second example: exposure of rats to polytetrafluoroethylene (PTFE) fume. PTFE fume (generated by heating PTFE) has long been known to be of high acute toxicity to birds and mammals, including humans (Cavagna et al. 1961; Coleman et al. 1968; Griffith et al. 1973; Nuttall et al. 1964; Waritz and Kwon 1968). Analysis of these fumes revealed the nanosized nature of the particles generated by heating PTFE to about 480°C, with a count median diameter (CMD) of 18 nm. They were highly toxic to rats, causing severe acute lung injury with high mortality within 4 hr after a 15-min inhalation exposure to 50 μg/m^3^ (Oberdörster et al. 1995). This short exposure resulted in an estimated deposited dose in the alveolar regions of only approximately 60 ng. In humans, acute lung injury, known as polymer fume fever, can result from exposures to PTFE fumes (Auclair et al. 1983; Goldstein et al. 1987; Lee et al. 1997; Williams et al. 1974; Woo et al. 2001). Additional rat studies showed that the gas phase alone was not acutely toxic and that aging of the PTFE fume particles for 3 min increased their particle size to > 100 nm because of accumulation, which resulted in a loss of toxicity (Johnston et al. 2000). However, it is most likely that changes in particle surface chemistry during the aging period contributed to this loss of toxicity, and that this is not just an effect of the accumulated larger particle size [Supplemental Material available online (http://ehp.niehs.nih.gov/members/2005/7339/supplemental.pdf)].

These examples seem to represent the extremes of NSPs in terms of pulmonary toxicity, one (TiO_2_) being rather benign yet still inducing significantly greater inflammatory effects on a mass basis than fine particles of the same chemical makeup, the other (PTFE fumes) inducing very high acute toxicity, possibly related to reactive groups on the large surface per unit mass.

Engineered nanomaterials can have very different shapes, for example, spheres, fibers, tubes, rings, and planes. Toxicologic studies of spherical and fibrous particles have well established that natural (e.g., asbestos) and man-made (e.g., biopersistent vitreous) fibers are associated with increased risks of pulmonary fibrosis and cancer after prolonged exposures [Greim et al. 2001; International Agency for Research on Cancer (IARC) 2002]. Critical parameters are the three Ds: dose, dimension, and durability of the fibers. Fibers are defined as elongated structures with a diameter-to-length ratio (aspect ratio) of 1:3 or greater and with a length of > 5 μm and diameter ≤3 μm [World Health Organization (WHO) 1985]. Carbon nanotubes have aspect ratios of up to ≥100, and length can exceed 5 μm with diameters ranging from 0.7 to 1.5 nm for single-walled nanotubes, and 2 to 50 nm for multiwalled nanotubes. Results from three studies using intratracheal dosing of carbon nanotubes in rodents indicate significant acute inflammatory pulmonary effects that either subsided in rats (Warheit et al. 2004) or were more persistent in mice (Lam et al. 2004; Shvedova et al. 2004b). Administered doses were very high, ranging from 1 to 5 mg/kg in rats; in mice doses ranged from 3.3 to 16.6 mg/kg (Lam et al. 2004) or somewhat lower, from 0.3 to 1.3 mg/kg (Shvedova et al. 2004a). Granuloma formation as a normal foreign body response of the lung to high doses of a persistent particulate material was a consistent finding in these studies. Metal impurities (e.g., iron) from the nanotube generation process may also have contributed to the observed effects. Although these *in vivo* first studies revealed high acute effects, including mortality, this was explained by the large doses of the instilled highly aggregated nanotubes that caused death by obstructing the airways and should not be considered a nanotube effect per se (Warheit et al. 2004). *In vitro* studies with carbon nanotubes also reported significant effects. Dosing keratinocytes and bronchial epithelial cells *in vitro* with single-walled carbon nanotubes (SWNTs) resulted in oxidative stress, as evidenced by the formation of free radicals, accumulation of peroxidative products, and depletion of cell antioxidants (Shvedova et al. 2004a, 2004b). Multiwalled carbon nanotubes (MWNTs) showed pro-inflammatory effects and were internalized in keratinocytes (Monteiro-Riviere et al. 2005). Again, the relatively high doses applied in these studies need to be considered when discussing the relevancy of these findings for *in vivo* exposures. A most recent study in macrophages comparing SWNTs and MWNTs with C_60_ fullerenes found a cytotoxicity ranking on a mass basis in the order SWNT > MWNT > C_60_. Profound cytotoxicity (mitochondrial function, cell morphology, phagocytic function) was seen for SWNTs, even at a low concentration of 0.38 μg/cm^2^. The possible contribution of metal impurities of the nanotubes still needs to be assessed. Therefore, whether the generally recognized principles of fiber toxicology apply to these nanofiber structures needs still to be determined (Huczko et al. 2001).

Future studies should be designed to investigate both effects and also the fate of nanotubes after deposition in the respiratory tract, preferentially by inhalation using well-dispersed (singlet) airborne nanotubes. In order to design the studies using appropriate dosing, it is necessary to assess the likelihood and degree of human exposure. It is of utmost importance to characterize human exposures in terms of the physicochemical nature, the aggregation state, and concentration (number, mass, surface area) of engineered nanomaterials and perform animal and *in vitro* studies accordingly. If using direct instillation into the lower respiratory tract, a large range of doses, which include expected realistic exposures of appropriately prepared samples, needs to be considered [Supplemental Material available online (http://ehp.niehs.nih.gov/members/2005/7339/supplemental.pdf)].

#### Ecotoxicologic studies.

Studies have been carried out to date on only a few species that have been accepted by regulatory agencies as models for defining ecotoxicologic effects. Tests with uncoated, water-soluble, colloidal fullerenes (nC_60_) show that the 48-hr LC_50_ (median lethal concentration) in *Daphnia magna* is 800 ppb (Oberdörster E 2004b), using standard U.S. EPA protocols (U.S. EPA 1994). In largemouth bass (*Micropterus salmoides*), although no mortality was seen, lipid peroxidation in the brain and glutathione depletion in the gill were observed after exposure to 0.5 ppm nC_60_ for 48 hr (Oberdörster E 2004a). There are several hypotheses as to how lipid damage may have occurred in the brain, including direct redox activity by fullerenes reaching the brain via circulation or axonal translocation (see also “Disposition of NSPs in the respiratory tract,” below) and dissolving into the lipid-rich brain tissue; oxyradical production by microglia; or reactive fullerene metabolites may be produced by cytochrome P450 metabolism. Initial follow-up studies using suppressive subtractive hybridization of pooled control fish versus pooled 0.5-ppm–exposed fish liver mRNA were also performed. Proteins related to immune responses and tissue repair were up-regulated, and several proteins related to homeostatic control and immune control were down-regulated. A cytochrome P450 (CYP2K4) involved in lipid metabolism was up-regulated [Supplemental Material available online (http://ehp.niehs.nih.gov/members/2005/7339/supplemental.pdf)]. In addition to these biochemical and molecular-level changes in fish, bactericidal properties of fullerenes have also been reported and are being explored as potential new antimicrobial agents (Yamakoshi et al. 2003). Engineered nanomaterials used as antimicrobials may shift microbial communities if they are released into the environment via effluents. As we know from anthropogenic endocrine-disrupting compounds, interference of signaling between nitrogen-fixing bacteria and their plant hosts could be extremely harmful both ecologically and economically in terms of crop production (Fox et al. 2001).

**Figure 5:**
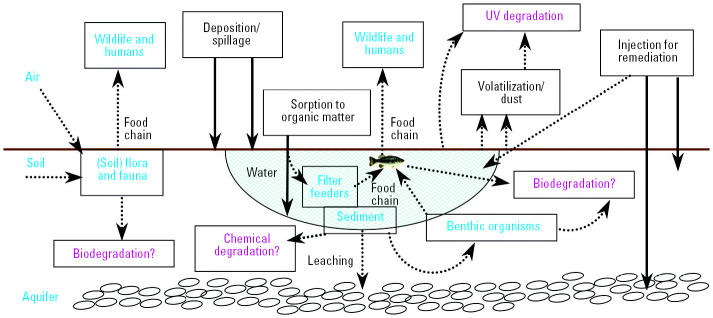
Routes of exposure, uptake, distribution, and degradation of NSPs in the environment. Solid lines indicate routes that have been demonstrated in the laboratory or field or that are currently in use (remediation). Magenta lettering indicates possible degradation routes, and blue lettering indicates possible sinks and sources of NSPs.

Aqueous fullerenes and coated SWNTs are stable in salt solutions (Cheng et al. 2004; Warheit et al. 2004), cell culture media (Lu et al. 2004; Sayes et al. 2004), reconstituted hard water, and MilliQ water (Dieckmann et al. 2003; Oberdörster E 2004a, 2004b). NSPs will tend to sorb onto sediment and soil particles and be immobilized because of their high surface area:mass ratio (Lecoanet and Wiesner 2004). Biologic transport would occur from ingested sediments, and one would expect movement of nanomaterials through the food chain (Figure 5).

To make engineered nanomaterials more biocompatible, both surface coatings and covalent surface modifications have been incorporated. Some studies have shown that both the surface coating and the covalent modifications can be weathered either by exposure to the oxygen in air or by ultraviolet (UV) irradiation for 1–4 hr (Derfus et al. 2004; Rancan et al. 2002). Therefore, although coatings and surface modifications may be critically important in drug-delivery devices, the likelihood of weathering under environmental conditions makes it important to study toxicity under UV and air exposure conditions. Even coatings used in drug delivery of NPs may not be bio-persistent or could be metabolized to expose the core NP material [Supplemental Material available online (http://ehp.niehs.nih.gov/members/2005/7339/supplemental.pdf)].

#### Reactive oxygen species mechanisms of NSP toxicity.

Both *in vivo* and *in vitro*, NSPs of various chemistries have been shown to create reactive oxygen species (ROS). ROS production has been found in NPs as diverse as C_60_ fullerenes, SWNTs, quantum dots, and UFPs, especially under concomitant exposure to light, UV, or transition metals (Brown et al. 2000, 2001; Derfus et al. 2004; Joo et al. 2004; Li et al. 2003; Nagaveni et al. 2004; Oberdörster E 2004a; Rancan et al. 2002; Sayes et al. 2004; Shvedova et al. 2004a, 2004b; Wilson et al. 2002; Yamakoshi et al. 2003). It has been demonstrated that NSPs of various sizes and various chemical compositions preferentially mobilize to mitochondria (de Lorenzo 1970; Foley et al. 2002; Gopinath et al. 1978; Li et al. 2003; Rodoslav et al. 2003). Because mitochondria are redox active organelles, there is a likelihood of altering ROS production and thereby overloading or interfering with antioxidant defenses (Figure 3).

**Figure 6:**
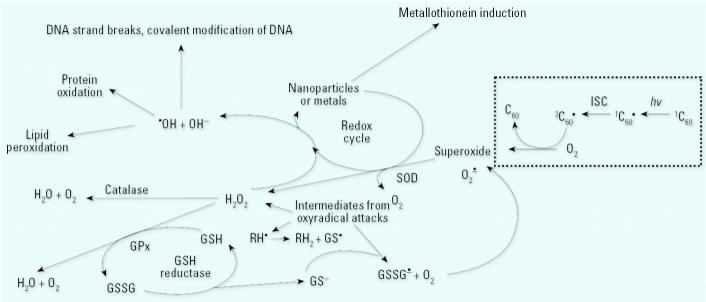
NPs have been shown to release oxyradicals [pictured here is the mechanism of C_60_ as determined by Yamakoshi et al. (2003)], which can interact with the antioxidant defense system. Abbreviations: GPx, glutathione peroxidase; GSH, reduced glutathione; GSSG, oxidized glutathione; ISC, intersystem crossing; R, any organic molecule; SOD, superoxide dismutase. In addition to fullerenes, metals such as cadmium, iron, or nickel quantum dots, or iron from SWNT manufacturing, could also act in Fenton-type reactions. Phase II biotransformation, ascorbic acid, vitamin E, beta carotene, and other interactions are not shown.

Figure 6 diagrams some of the antioxidant defense systems that occur in animals, and possible areas where NSPs may create oxyradicals. The C_60_ fullerene is shown as a model NP producing superoxide, as has been shown by Yamakoshi et al. (2003). The exact mechanism by which each of these diverse NPs cause ROS is not yet fully understood, but suggested mechanisms include *a*) photo excitation of fullerenes and SWNTs, causing intersystem crossing to create free electrons; *b*) metabolism of NPs to create redox active intermediates, especially if metabolism is via cytochrome P450s; and *c*) inflammation responses *in vivo* that may cause oxyradical release by macrophages. Other mechanisms will likely emerge as studies on NP toxicity continue.

The small size and respective large specific surface area of NPs, like those of ambient airborne UFPs, give them unique properties with respect to a potential to cause adverse effects. Certainly, as shown in studies with UFPs, chemical composition and other particle parameters are additional important effect modifiers. Results from these studies will therefore serve as a basis for future studies in the field of nanotoxicology, for example, the propensity of NSPs to translocate across cell layers and along neuronal pathways (see “Disposition of NSPs in the respiratory tract” below).

#### Exposure dose–response considerations.

A careful evaluation of exposure–dose–response relationships is critical to the toxicologic assessment of NSPs. This includes not only questions about the dosemetric—mass, number, or surface of the particles, as discussed above—but most important, also the relevance of dose levels. For example, it is tempting, and a continual practice, to dose primary cells or cell lines *in vitro* with very high doses without any consideration or discussion of realistic *in vivo* exposures; for instance, 100 μg NSPs/mL culture medium—labeled as a low dose—is extremely high and is unlikely to be encountered *in vivo*. Likewise, intratracheal instillations of several hundred micrograms into a rat does not resemble a relevant *in vivo* inhalation exposure; both dose and dose rate cause high bolus dose artifacts. Although such studies may be used in a first proof-of-principle approach, it is mandatory to follow up and validate results using orders of magnitude lower concentrations resembling realistic *in vivo* exposures, including worst-case scenarios. The 500-year-old phrase “the dose makes the poison” can also be paraphrased as “the dose makes the mechanism.” The mechanistic pathways that operate at low realistic doses are likely to be different from those operating at very high doses when the cell’s or organism’s defenses are overwhelmed.

**Figure 7:**
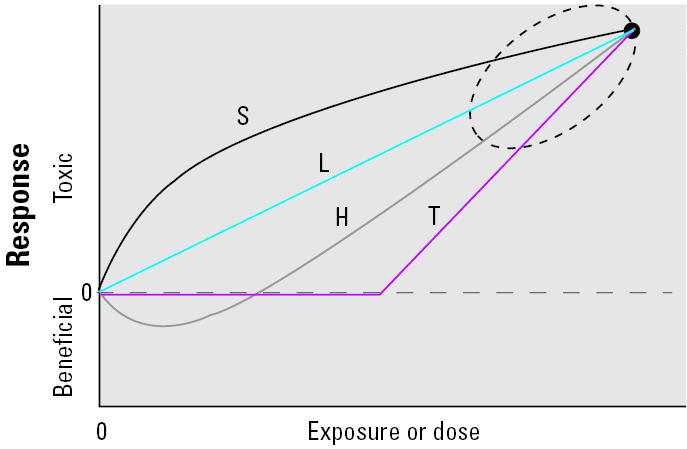
Some basic shapes of exposure–response or dose–response relationships. Abbreviations: H, hormetic (biphasic); L, linear (no threshold); S, supralinear; T, threshold. Prerequisites for establishing these relationships for NSPs from *in vitro* or *in vivo* studies include a sufficient number of data points, that is, over a wide range of exposure concentrations or doses; knowledge about exposure levels; and information about correlation of exposure with doses at the organismal or cellular level (an exposure is not a dose). Dose–response curves of different shapes can be extrapolated when only response data at high dose levels (indicated by dashed oval) are available. Lack of data in the low—oftentimes the most relevant—dose range can result in severe misinterpretation if a threshold or even a hormetic response is present. Consideration also needs to be given to the likelihood that the shape or slope of exposure–dose–response relationships change for susceptible parts of the population.

Therefore, *in vivo* and *in vitro* studies will provide useful data on the toxicity and mode of action of NSPs only if justifiable concentrations/doses are considered when designing such studies. This approach is particularly important for the proper identification of the dose–response curve. When data are generated only at high concentrations/doses, it is difficult to determine whether the dose–response curve in question is best described by a linear (no threshold), supralinear, threshold, or hormetic model (Figure 7). Study designs should include doses that most closely reflect the expected exposure levels. A critical gap that urgently needs to be filled in this context is the complete lack of data for human or environmental exposure levels of NSPs. Furthermore, some knowledge about the biokinetics of NSPs is required in order to estimate appropriate doses. Do specific NPs reach certain target sites? If so, what are the doses, dose rates, and their persistence? Further, although it may be tempting to extrapolate from *in vitro* results to an *in vivo* risk assessment, it is important to keep in mind that *in vitro* tests are most useful in providing information on mechanistic processes and in elucidating mechanisms/mode of actions suggested by studies in whole animals. A combination of *in vitro* and *in vivo* studies with relevant dose levels will be most useful in identifying the potential hazards of NPs, and a thorough discussion and justification of selected dose levels should be mandatory.

## Portals of Entry and Target Tissues

Most of the toxicity research on NSPs *in vivo* has been carried out in mammalian systems, with a focus on respiratory system exposures for testing the hypothesis that airborne UFPs cause significant health effects. With respect to NPs, other exposure routes, such as skin and GI tract, also need to be considered as potential portals of entry. Portal-of-entry–specific defense mechanisms protect the mammalian organism from harmful materials. However, these defenses may not always be as effective for NSPs, as is discussed below.

### Respiratory Tract

In order to appreciate what dose the organism receives when airborne particles are inhaled, information about their deposition as well as their subsequent fate is needed. Here we focus on the fate of inhaled nanosized materials both within the respiratory tract itself and translocated out of the respiratory tract. There are significant differences between NSPs and larger particles regarding their behavior during deposition and clearance in the respiratory tract [Supplemental Material available online (http://ehp.niehs.nih.gov/members/2005/7339/supplemental.pdf)].

#### Efficient deposition of inhaled NSPs.

**Figure 8:**
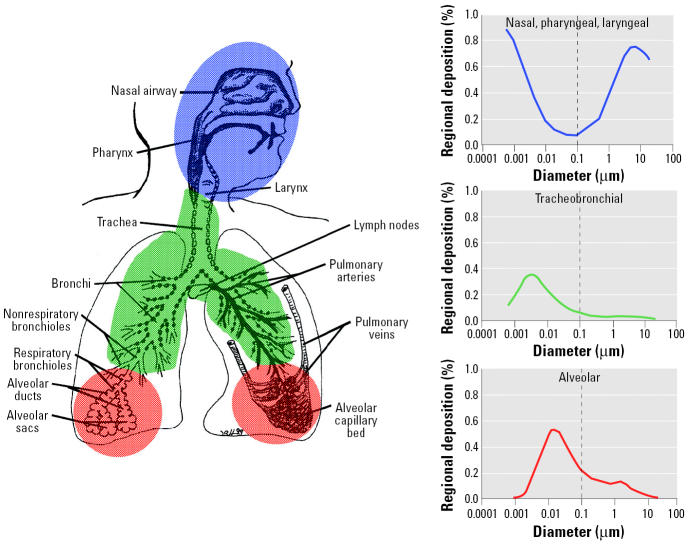
Predicted fractional deposition of inhaled particles in the nasopharyngeal, tracheobronchial, and alveolar region of the human respiratory tract during nose breathing. Based on data from the International Commission on Radiological Protection (1994). Drawing courtesy of J. Harkema.

The main mechanism for deposition of inhaled NSPs in the respiratory tract is diffusion due to displacement when they collide with air molecules. Other deposition mechanisms of importance for larger particles, such as inertial impaction, gravitational settling, and interception, do not contribute to NSP deposition, and electrostatic precipitation occurs only in cases where NSPs carry significant electric charges. Figure 8 shows the fractional deposition of inhaled particles in the nasopharyngeal, tracheobronchial, and alveolar regions of the human respiratory tract under conditions of nose breathing during rest, based on a predictive mathematical model (International Commission on Radiological Protection 1994). These predictions apply to particles that are inhaled as singlet particles of a given size and not as aggregates; the latter obviously will have larger particle size and different deposition site. In each of the three regions of the respiratory tract, significant amounts of a certain size of NSPs (1–100 nm) are deposited. For example, 90% of inhaled 1-nm particles are deposited in the nasopharyngeal compartment, only approximately 10% in the tracheobronchial region, and essentially none in the alveolar region. On the other hand, 5-nm particles show about equal deposition of approximately 30% of the inhaled particles in all three regions; 20-nm particles have the highest deposition efficiency in the alveolar region (~ 50%), whereas in tracheobronchial and nasopharyngeal regions this particle size deposits with approximately 15% efficiency. These different deposition efficiencies should have consequences for potential effects induced by inhaled NSPs of different sizes as well as for their disposition to extrapulmonary organs, as discussed further below.

#### Disposition of NSPs in the respiratory tract.

In the preceding section we summarized data demonstrating that inhaled NSPs of different sizes can target all three regions of the respiratory tract. Several defense mechanisms exist throughout the respiratory tract aimed at keeping the mucosal surfaces free from cell debris and particles deposited by inhalation. Several reviews describe the well-known classic clearance mechanisms and pathways for deposited particles (Kreyling and Scheuch 2000; Schlesinger et al. 1997; U.S. EPA 2004), so here we only briefly mention those mechanisms and point out specific differences that exist with respect to inhaled NSPs [Supplemental Material available online (http://ehp.niehs.nih.gov/members/2005/7339/supplemental.pdf)].

Once deposited, NSPs—in contrast to larger-sized particles—appear to translocate readily to extrapulmonary sites and reach other target organs by different transfer routes and mechanisms. One involves transcytosis across epithelia of the respiratory tract into the interstitium and access to the blood circulation directly or via lymphatics, resulting in distribution throughout the body. The other is a not generally recognized mechanism that appears to be distinct for NSPs and that involves their uptake by sensory nerve endings embedded in airway epithelia, followed by axonal translocation to ganglionic and CNS structures.

##### Classical clearance pathways.

**Figure 9:**
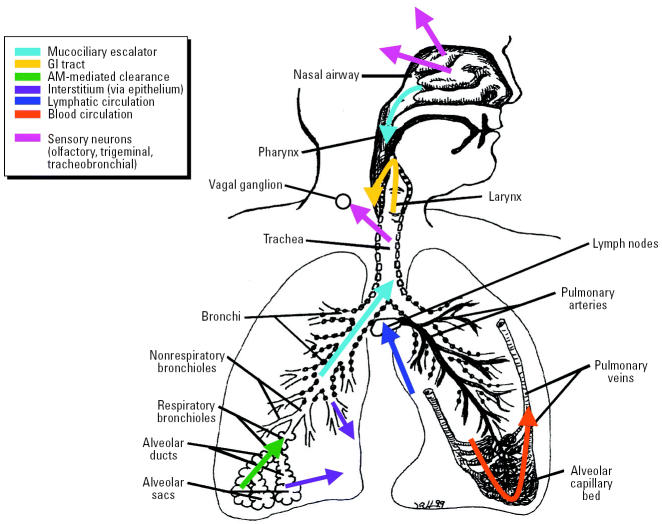
Pathways of particle clearance (disposition) in and out of the respiratory tract. There are significant differences between NSPs and larger particles for some of these pathways (see “Disposition of NSPs in the respiratory tract”). Drawing courtesy of J. Harkema.

The clearance of deposited particles in the respiratory tract is basically due to two processes (Table 3): *a*) physical translocation of particles by different mechanisms and *b*) chemical clearance processes. Leaching refers to loss of elements from a particle matrix (e.g., loss of sodium from asbestos fibers due to dissolution in intra-or extracellular milieu). Chemical dissolution is directed at biosoluble particles or components of particles that are either lipid soluble or soluble in intracellular and extracellular fluids. Solutes and soluble components can then undergo absorption and diffusion or binding to proteins and other subcellular structures and may be eventually cleared into blood and lymphatic circulation. Chemical clearance for biosoluble materials can happen at any location within the three regions of the respiratory tract, although to different degrees, depending on local extracellular and intracellular conditions (pH). In contrast, a number of diverse processes involving physical translocation of inhaled particles exist in the respiratory tract and are different in the three regions. Figure 9 summarizes these clearance processes for solid particles. As discussed further below, some of them show significant particle-size–dependent differences, making them uniquely effective for a certain particle size but very inefficient for other sizes.

The most prevalent mechanism for solid particle clearance in the alveolar region is mediated by alveolar macrophages, through phagocytosis of deposited particles. The success of macrophage–particle encounter appears to be facilitated by chemotactic attraction of alveolar macrophages to the site of particle deposition (Warheit et al. 1988). The chemotactic signal is most likely complement protein 5a (C5a), derived from activation of the complement cascade from serum proteins present on the alveolar surface (Warheit et al. 1986; Warheit and Hartsky 1993). This is followed by gradual movement of the macrophages with internalized particles toward the mucociliary escalator. The retention half-time of solid particles in the alveolar region based on this clearance mechanism is about 70 days in rats and up to 700 days in humans. The efficacy of this clearance mechanism depends highly on the efficiency of alveolar macrophages to “sense” deposited particles, move to the site of their deposition, and then phagocytize them. This process of phagocytosis of deposited particles takes place within a few hours, so by 6–12 hr after deposition essentially all of the particles are phagocytized by alveolar macrophages, to be cleared subsequently by the slow alveolar clearance mentioned above. However, it appears that there are significant particle-size–dependent differences in the cascade of events leading to effective alveolar macrophage-mediated clearance.

**Figure 10:**
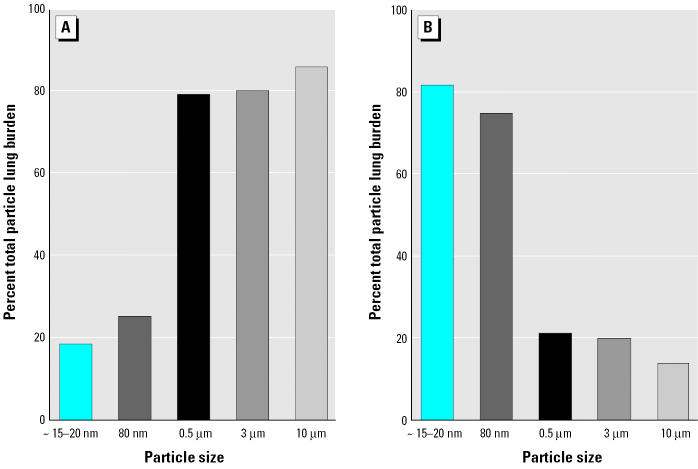
*In vivo* retention of inhaled nanosized and larger particles in alveolar macrophages (*A*) and in exhaustively lavaged lungs (epithelial and interstitial retention; *B*) 24 hr postexposure. The alveolar macrophage is the most important defense mechanism in the alveolar region for fine and coarse particles, yet inhaled singlet NSPs are not efficiently phagocytized by alveolar macrophages.

Figure 10 displays results of several studies in which rats were exposed to different-sized particles (for the 3- and 10-μm particles, 10-μg and 40-μg polystyrene beads, respectively, were instilled intratracheally) (Kreyling et al. 2002; Oberdörster et al. 1992b, 2000; Semmler et al. 2004). Twenty-four hours later, the lungs of the animals were lavaged repeatedly, retrieving about 80% of the total macrophages as determined in earlier lavage experiments (Ferin et al. 1991). As shown in Figure 10, approximately 80% of 0.5-, 3-, and 10-μm particles could be retrieved with the macrophages, whereas only approximately 20% of nanosized 15–20-nm and 80-nm particles could be lavaged with the macrophages. In effect, approximately 80% of the UFPs were retained in the lavaged lung after exhaustive lavage, whereas approximately 20% of the larger particles > 0.5 μm remained in the lavaged lung. This indicates that NSPs either were in epithelial cells or had further translocated to the interstitium [Supplemental Material available online (http://ehp.niehs.nih.gov/members/2005/7339/supplemental.pdf)].

##### Epithelial translocation.

Because of the apparent inefficiency of alveolar macrophage phagocytosis of NSPs, one might expect that these particles interact instead with epithelial cells. Indeed, results from several studies show that NSPs deposited in the respiratory tract readily gain access to epithelial and interstitial sites. This was also shown in studies with ultrafine PTFE fumes: shortly after a 15-min exposure, the fluorine-containing particles could be found in interstitial and submucosal sites of the conducting airways as well as in the interstitium of the lung periphery close to the pleura (Oberdörster G 2000). Such interstitial translocation represents a shift in target site away from the alveolar space to the interstitium, potentially causing direct particle-induced effects there.

In a study evaluating the pulmonary inflammatory response of TiO_2_ particles, ranging from NP TiO_2_ to pigment-grade TiO_2_ (12–250 nm), a surprising finding was that, 24 hr after intratracheal instillation of different doses, higher doses induced a lower effect (Oberdörster et al. 1992a). This was explained by the additional finding that at the higher doses (expressed as particle surface area) of the nanosized TiO_2_, ≥50% had reached the pulmonary interstitium, causing a shift of the inflammatory cell response from the alveolar space to the interstitium [Supplemental Material available online (http://ehp.niehs.nih.gov/members/2005/7339/supplemental.pdf)]. The smaller particle size of 12 and 20 nm versus 220 and 250 nm also means that the administered particle number was more than three orders of magnitude higher for the NSPs, a factor that seems to be an important determinant for particle translocation across the alveolar epithelium, as are the delivered total dose and the dose rate (Ferin et al. 1992). Because interstitial translocation of fine particles across the alveolar epithelium is more prominent in larger species (dogs, nonhuman primates) than in rodents (Kreyling and Scheuch 2000; Nikula et al. 1997), it is reasonable to assume that the high translocation of NSPs observed in rats occurs in humans as well [Supplemental Material available online (http://ehp.niehs.nih.gov/members/2005/7339/supplemental.pdf)].

##### Translocation to the circulatory system.

Once the particles have reached pulmonary interstitial sites, uptake into the blood circulation, in addition to lymphatic pathways, can occur; again, this pathway is dependent on particle size, favoring NSPs. Berry et al. (1977) were the first to describe translocation of NSPs across the alveolar epithelium using intratracheal instillations of 30-nm gold particles in rats. Within 30 min postexposure, they found large amounts of these particles in platelets of pulmonary capillaries; the researchers suggested that this is an elimination pathway for inhaled particles that is significant for transporting the smallest air pollutant particles—in particular, particles of tobacco smoke—to distant organs. They also hypothesized that this “might predispose to platelet aggregation with formation of microthrombi atheromatous plaques” (Berry et al. 1977).

**Figure 11:**
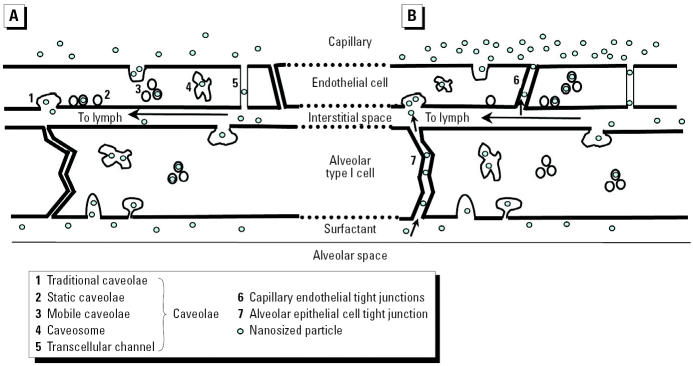
Different forms of caveolae and cellular tight junctions function as translocation mechanisms across cell layers. Depending on particle surface chemistry, NSPs have been shown to transcytose across alveolar type I epithelial cells and capillary endothelial cells (Table 4), but not via cellular tight junctions in the healthy state (*A*). However, in a compromised or disease state (e.g., endotoxin exposure; *B*) translocation across widened tight junction occurs as well (Heckel et al. 2004). This indicates that assessing potential effects of NSPs in the compromised state is an important component of nanotoxicology. Adapted from Cohen et al. (2004).

Since then, a number of studies with different particle types have confirmed the existence of this translocation pathway, as summarized in Table 4. Collectively, these studies indicate that particle size and surface chemistry (coating), and possibly charge, govern translocation across epithelial and endothelial cell layers. In particular, the studies summarized by Mehta et al. (2004) and those performed by Heckel et al. (2004) using intravenous administration of albumin-coated gold nanoparticles in rodents demonstrated receptor-mediated transcytosis (albumin-binding proteins) via caveolae (Figure 11). These 50–100 nm vesicles, first described by Simionescu et al. (1975), form from indentations of the plasmalemma and are coated with the caveolin-1 protein. Albumin, as the most abundant protein in plasma and interstitium, appears to facilitate NP endocytosis, as does lecithin, a phospholipid: even 240-nm polystyrene particles translocated across the alveolo-capillary barrier when coated with lecithin, whereas uncoated particles did not (Kato et al. 2003). The presence of both albumin and phospholipids in alveolar epithelial lining fluid may, therefore, be important constituents for facilitated epithelial cell uptake of NSPs after deposition in the alveolar space.

Rejman et al. (2004) reviewed a number of different endocytic pathways for internalization of a variety of substances, including phagocytosis, macropinocytosis, clathrin-mediated endocytosis, and caveolae-mediated endocytosis. They found in nonphagocytic cells *in vitro* that internalization via clathrin-coated pits prevailed for latex microspheres < 200 nm, whereas with increasing size up to 500 nm, caveolae became the predominant pathway. However, as shown in Table 4, surface coating of NSPs with albumin clearly causes even the smallest particles to be internalized via caveolae. The presence of caveolae on cells differs: they are abundant in lung capillaries and alveolar type l cells but not in brain capillaries (Gumbleton 2001). In the lung, during inspiratory expansion and expiratory contraction of the alveolar walls, caveolae with openings around 40 nm disappear and reappear, forming vesicles that are thought to function as transport pathways across the cells for macromolecules (Patton 1996). Knowledge from virology about cell entry of biologic NSPs (viruses) via clathrin-coated pits and caveolae mechanisms should also be considered (Smith and Helenius 2004) and can shed light on the mechanism by which engineered NPs may enter cells and interact with subcellular structures.

Evidence in humans for the translocation of inhaled NSPs into the blood circulation is ambiguous, with one study showing rapid appearance in the blood and significant accumulation of label in the liver of humans inhaling ^99^Tc-labeled 20-nm carbon particles (Nemmar et al. 2002a), whereas another study using the same labeled particles reported no such accumulation (Brown et al. 2002). Taking into consideration all of the evidence from animal and human studies for alveolar translocation of NSPs, it is likely that this pathway also exists in humans; however, the extent of extrapulmonary translocation is highly dependent on particle surface characteristics/chemistry, in addition to particle size. Translocation to the blood circulation could provide a mechanism for a direct particle effect on the cardiovascular system as an explanation for epidemiologic findings of cardiovascular effects associated with inhaled ambient UFPs (Pekkanen et al. 2002; Wichmann et al. 2000) and for results of clinical studies showing vascular responses to inhaled elemental carbon UFPs (Pietropaoli et al. 2004). In addition to direct alveolar translocation of NSPs, cardiovascular effects may also be the corollary of a sequence of events starting with particle-induced alveolar inflammation initiating a systemic acute phase response with changes in blood coagulability and resulting in cardiovascular effects (Seaton et al. 1995).

Once NSPs have translocated to the blood circulation, they can be distributed throughout the body. The liver is the major distribution site via uptake by Kupffer cells, followed by the spleen as another organ of the reticulo-endothelial system, although coating with polyethylene glycol (PEG) almost completely prevents hepatic and splenic localization so that other organs can be targeted (Akerman et al. 2002). Distribution to heart, kidney, and immune-modulating organs (spleen, bone marrow) has been reported. For example, several types of NPs, ranging from 10 to 240 nm, localized to a significant degree in bone marrow after intravenous injection into mice (Table 5). Such target specificity may be extremely valuable for drug delivery; for example, drug delivery to the CNS via blood-borne NPs requires NP surface modifications in order to facilitate translocation across the tight blood–brain barrier via specific receptors (e.g., apolipoprotein coating for LDL-receptor–mediated endocytosis in brain capillaries) (Kreuter 2001, 2004; Kreuter et al. 2002). Such highly desirable properties of NPs must be carefully weighed against potential adverse cellular responses of targeted NP drug delivery, and a rigorous toxicologic assessment is mandatory [Supplemental Material available online (http://ehp.niehs.nih.gov/members/2005/7339/supplemental.pdf)].

##### Neuronal uptake and translocation.

**Figure 12:**
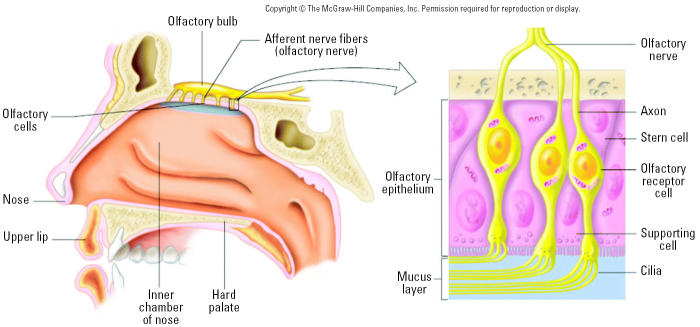
Close proximity of olfactory mucosa to olfactory bulb of the CNS. Inhaled NSP[s], especially below 10 nm, deposit efficiently on the olfactory mucosa by diffusion, similar to airborne “smell” molecules which deposit in this area of olfactory dendritic cilia. Subsequent uptake and translocation of solid NSP[s] along axons of the olfactory nerve has been demonstrated in non-human primates and rodents. Surface chemistry of the particles may influence their neuronal translocation. Copyright © the McGraw-Hill Companies, Inc. Reproduced from Widmaier et al. (2004) with permission from McGraw-Hill.

A translocation pathway for solid particles in the respiratory tract involving neuronal axons is apparently specific for NSPs. Respective studies are summarized in Table 6. This pathway was described > 60 years ago, yet it has received little or no attention from toxicologists. This pathway, shown in Figure 9 for the nasal and tracheobronchial regions, comprises sensory nerve endings of the olfactory and the trigeminus nerves and an intricate network of sensory nerve endings in the tracheobronchial region. These early studies concerned a large series of studies with 30-nm polio virus intranasally instilled into chimpanzees and rhesus monkeys (Bodian and Howe 1941a, 1941b; Howe and Bodian 1940). Their studies revealed that the olfactory nerve and olfactory bulbs are, indeed, portals of entry to the CNS for intranasally instilled nanosized polio virus particles, which could subsequently be recovered from the olfactory bulbs. The close proximity of nasal olfactory mucosa and olfactory bulb requires only a short distance to be covered by neuronal transport (Figure 12). Bodian and Howe (1941b) determined the transport velocity of the virus in the axoplasm of axons to be 2.4 mm/hr, which is very well in agreement with neuronal transport velocities measured later by Adams and Bray (1983) for solid particles (up to 500 nm) directly microinjected into giant axons of crabs, and by de Lorenzo (1970) for silver-coated colloidal gold (50 nm) in squirrel monkeys.

The de Lorenzo (1970) study demonstrated in squirrel monkeys that intranasally instilled silver-coated colloidal gold particles (50 nm) translocated anterogradely in the axons of the olfactory nerves to the olfactory bulbs. The 50-nm gold particles even crossed synapses in the olfactory glomerulus to reach mitral cell dendrites within 1 hr after intranasal instillation. An interesting finding in this study—and important for potential adverse effects—was that the NSPs in the olfactory bulb were no longer freely distributed in the cytoplasm but were preferentially located in mitochondria (see also “Reactive oxygen species mechanisms of NSP toxicity,” above).

**Figure 13:**
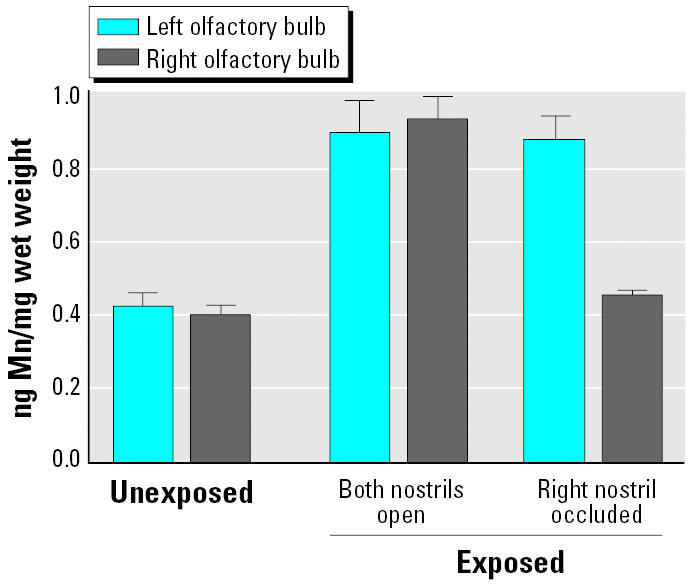
Occlusion of the right nostril of rats during 6-hr inhalation of nanosized MnO_2_ particles (~ 30 nm CMD, ~ 450 μg/m^3^) resulted in accumulation of Mn only in the left olfactory bulb only at 24 hr after dosing. Data are mean ± SD. Data from Feikert et al. (2004).

Newer studies indicated that this translocation pathway is also operational for inhaled NSPs. Inhalation of elemental ^13^C UFPs (CMD = 35 nm) resulted in a significant increase of ^13^C in the olfactory bulb on day 1, which increased further throughout day 7 post-exposure (Oberdörster et al. 2004). Results of another inhalation study with solid nanosized (CMD = 30 nm) manganese oxide (MnO_2_) particles in rats showed after a 12-day exposure a more than 3.5-fold significant increase of Mn in the olfactory bulb, compared with only a doubling of Mn in the lung. When one nostril was occluded during a 6-hr exposure, Mn accumulation in the olfactory bulb was restricted to the side of the open nostril only (Figure 13) (Feikert et al. 2004). This result contrasts with 15-day inhalation of larger-sized MnO_2_ particles in rats (1.3 and 18 μm mass median aerodynamic diameter) where no significant increases in olfactory Mn was found (Fechter et al. 2002). This was to be expected given that the individual axons of the fila olfactoria (forming the olfactory nerve) are only 100–200 nm in diameter (de Lorenzo 1957; Plattig 1989).

Collectively, these studies point out that the olfactory nerve pathway should also be considered a portal of entry to the CNS for humans under conditions of environmental and occupational exposures to airborne NSPs. However, there are important differences between rodents and humans. The olfactory mucosa of the human nose comprises only 5% of the total nasal mucosal surface as opposed to 50% in rats—which in addition are obligatory nose breathers (Table 7). One can argue that the olfactory route may therefore be an important transfer route to the CNS for inhaled NSPs in animals with a well-developed olfaction system, yet at the same time its importance for humans with a more rudimentary olfactory system can be questioned. However, estimates using a predictive particle deposition model and data from Table 7 show that concentrations of 20-nm translocated particles in the human olfactory bulb can, indeed, be 1.6–10 times greater than in rats [Supplemental Material available online (http://ehp.niehs.nih.gov/members/2005/7339/supplemental.pdf)].

Translocation into deeper brain structures may possibly occur as well, as shown in rats for soluble Mn (Gianutsos et al. 1997), but requires further confirmatory studies with respect to solid NSPs. Further evidence for movement of NSPs along axons and dendrites in humans is provided by knowledge accumulated by virologists who have long understood the movement of human meningitis virus through olfactory and trigeminal neurons and, similarly, herpes virus movement up and down the trigeminal neuron to trigger outbreaks of herpes cold sores in humans (Kennedy and Chaudhuri 2002; Terasaki et al. 1997).

There are additional neuronal translocation pathways for solid NSPs via the trigeminus nerve and tracheobronchial sensory nerves (Table 6). A study by Hunter and Dey (1998) in rats demonstrated the translocation of intranasally instilled rhodamine-labeled microspheres (20–200 nm) to the trigeminal ganglion inside the cranium via uptake into the ophthalmic and maxillary branches of the trigeminus nerve that supplies sensory nerve endings throughout the nasal mucosa. In another study, Hunter and Undem (1999) instilled the same microparticles intratracheally into guinea pigs; they found neuronal translocation of these solid microparticles to the ganglion nodosum in the neck area that is networked into the vagal system. This finding may be relevant for ambient UFPs because it can be hypothesized that cardiovascular effects associated with ambient particles in epidemiologic studies (Utell et al. 2002) are in part due to direct effects of translocated UFPs on the autonomic nervous system via sensory nerves in the respiratory tract.

In the context of potential CNS effects of air pollution, including ambient UFPs, two recent studies with exposures of mice to concentrated ambient fine particles and UFPs should be mentioned. Campbell et al. (2005) and Veronesi et al. (in press) found significant increases of tumor necrosis factor-α or decreases in dopaminergic neurons, supporting the hypothesis of ambient PM causing neuro-degenerative disease. A study by Calderon-Garcidueñas et al. (2002) may also point to an interesting link between air pollution and CNS effects: these authors described significant inflammatory or neurodegenerative changes in the olfactory mucosa, olfactory bulb, and cortical and subcortical brain structures in dogs from a heavily polluted area in Mexico City, whereas these changes were not seen in dogs from a less-polluted rural control city. However, whether direct effects of airborne UFPs are the cause of these effects remains to be determined.

Although the existence of neuronal translocation of NSPs has been well established, size alone is only one particle parameter governing this process. Surface characteristics of NSPs (chemistry, charge, shape, aggregation) are essential determinants as well, and it should not be assumed that all NSPs, when inhaled, will be distributed by the mechanism described here. It should be kept in mind, however, that the unique biokinetic behavior of NSPs—cellular endocytosis, transcytosis, neuronal and circulatory translocation and distribution—which makes them desirable for medical therapeutic or diagnostic applications—may be associated with potential toxicity. For example, NP-facilitated drug delivery to the CNS raises the question of the fate of NPs after their translocation to specific cell types or to subcellular structures in the brain. For example, does mitochondrial localization induce oxidative stress? How persistent is the coating or the core of the NPs? A respective safety evaluation is key [Supplemental Material available online (http://ehp.niehs.nih.gov/members/2005/7339/supplemental.pdf)].

### Exposure via GI Tract and Skin

NSPs cleared from the respiratory tract via the mucociliary escalator can subsequently be ingested into the GI tract. Alternatively, nanomaterials can be ingested directly, for example, if contained in food or water or if used in cosmetics or as drugs or drug delivery devices. Only a few studies have investigated the uptake and disposition of nanomaterials by the GI tract, and most have shown that NSPs pass through the GI tract and are eliminated rapidly. In rats dosed orally with radiolabeled functionalized C_60_ fullerenes, water solubilized using PEG and albumin (18 kBq in 100 μL), 98% were cleared in the feces within 48 hr, whereas the rest was eliminated via urine, indicating some uptake into the blood circulation (Yamago et al. 1995). In contrast, in this same study, 90% of the same radiolabeled fullerenes administered intravenously (9.6 kBq in ~ 50 μL or 14–18 kBq in 215 μL) were retained after 1 week, with most (73–80%, depending on time course) found in the liver. Studies by Kreyling and colleagues (Kreyling et al. 2002; Semmler et al. 2004) using ultrafine ^192^Ir did not show significant uptake in the GI tract, whereas earlier studies with larger TiO_2_ particles (150–500 nm) found uptake into the blood and movement to the liver (Böckmann et al. 2000; Jani et al. 1994). Likely there are differences in GI tract uptake dependent on both particle surface chemistry and particle size. Indeed, after oral dosing in rats, Jani et al. (1990) found a particle-size–dependent uptake of polystyrene particles (ranging from 50 to 3,000 nm) by the GI mucosa. This uptake (6.6% of the administered 50 nm, 5.8% of the 100 nm NSP, 0.8% of 1 μm particles, and 0% for 3 μm particles) was mainly via the Peyer's patches with translocation into the mesenteric lymph and then to systemic organs (i.e., liver, spleen, blood, bone marrow, and kidney).

**Figure 14:**
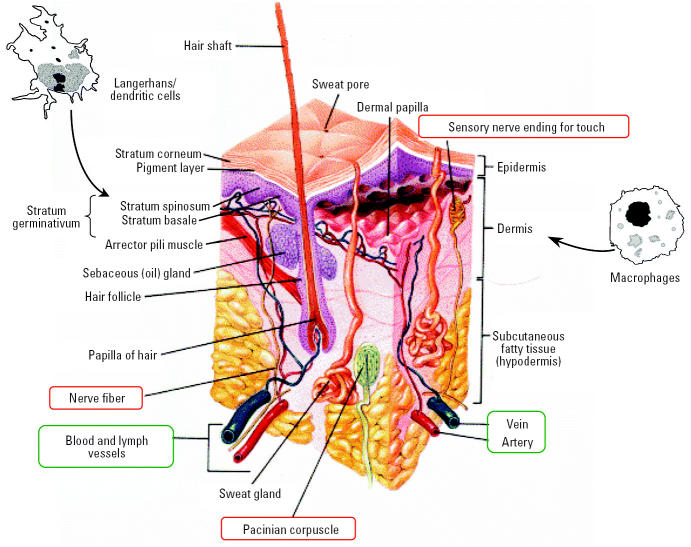
The epidermis represents a tight barrier against NSP penetration. Quantitatively, dermal translocation will therefore be minimal or nonexistent under normal conditions but increases in areas of skin flexing (Tinkle et al. 2003) and broken skin. Once in the dermis, lymphatic uptake is a major translocation route, likely facilitated by uptake in dendritic cells (epidermis) and macrophages; other potential pathways may include the dense networks of blood circulation and sensory nerves in the dermis. Adapted from Essential Day Spa (2005) with permission from www.essentialdayspa.com.

A potentially important uptake route is through dermal exposure. The epidermis, consisting of the outer horny layer (stratum corneum), the prickle cell layer (stratum spinosum), and basal cell layer (stratum basale), forms a very tight protective layer for the underlying dermis (Figure 14). The dermis has a rich supply of blood and tissue macrophages, lymph vessels, dendritic cells (Langerhans, also in stratum spinosum of epidermis), and five different types of sensory nerve endings. Broken skin represents a readily available portal of entry even for larger (0.5–7 μm) particles, as evidenced by reports about accumulation of large amounts of soil particles in inguinal lymph nodes of people who often run or walk barefoot; this can be associated with elephantiatic lymphedema (podoconiosis; Corachan et al. 1988; Blundell et al. 1989). Tinkle et al. (2003) hypothesized that unbroken skin when flexed—as in wrist movements—would make the epidermis permeable for NSPs. They demonstrated in a proof-of-concept experiment that, indeed, flexing the skin, but not flat skin, resulted in penetration of even 1 μm fluorescent beads to the dermis. The follow-up question about access of particles in the dermis to the circulation is answered by the aforementioned reports of podoconiosis, that is, uptake into the lymphatic system and regional lymph nodes. Subsequent translocation of NSPs beyond lymph nodes to the blood circulation is likely to occur as well, as shown in studies with small asbestos fibers (Oberdörster et al. 1988).

Newer studies by Kim et al. (2004) in mice and pigs with intradermally injected near-infrared quantum dots confirmed that NPs, once in the dermis, will localize to regional lymph nodes, which makes these particles very useful for *in vivo* imaging. Likely transport mechanisms to the lymph nodes are skin macrophages and dendritic (Langerhans) cells (Ohl et al. 2004; Sato et al. 1998); this raises a question about potential modulation of immune responses, after interaction of these NP-containing macrophages and dendritic cells with T lymphocytes. For example, Chen et al. (1998) were able to raise antibodies in mice specific for C_60_ after intraperitoneal injections of C_60_ conjugated to thyroglobulin and serum albumin. Clearly, research is needed to determine whether and under what conditions NPs can be recognized by the immune system, following any route of uptake into the organism.

Another question relates to the potential of sensory skin nerves to take up and translocate NPs. Given that this mechanism has been demonstrated for the nasal and tracheobronchial regions of the respiratory tract, how likely is this to occur in the dermis layer of the skin with its dense supply of different types of sensory nerves? It may be conceivable, considering data on neuronal uptake and translocation of NSPs after intramuscular injection. For example, nanosized ferritin and iron-dextran, after injection into the tongue of mice, labeled the neurons of the hypoglossal nuclei, and injection of both of these NSPs into facial muscles of mice also resulted in synaptic uptake; cationized ferritin was also detected in cell bodies of facial neurons, indicating that electrical charge is of importance for incorporation into axons and axonal transport (Arvidson 1994; Malmgren et al. 1978; Olsson and Kristensson 1981). Other studies using intramuscular injection of ferritin (~ 112 nm), iron-dextran (11 or 21 nm), and gold protein (20–25 nm) NSPs also showed rapid penetration through the basal lamina into the synaptic clef of the neuromuscular junction, but this was restricted to only the smaller nanoparticles, implying that there may be a size-dependent penetration of the basal lamina with a threshold somewhere between 10 and 20 nm (Oldfors and Fardeau 1983).

Neuronal transport of NSPs along sensory skin nerves is well established for herpes virus. After passing through the skin—especially broken skin—the viruses are transported retrogradely along dendrites of sensory neurons to the dorsal root ganglion, where they remain dormant until a stress situation triggers antero-grade translocation along the dendrites back to the skin (Kennedy and Chaudhuri 2002; Terasaki et al. 1997). Future studies need to determine whether and to what degree such translocation along sensory skin neurons also occurs with NPs penetrating the epidermis.

## Risk Assessment

The lack of toxicology data on engineered NPs does not allow for adequate risk assessment. Because of this, some may even believe that engineered NPs are so risky that they call for a precautionary halt in NP-related research. However, the precautionary principle should not be used to stop research related to nanotechnology and NPs. Instead, we should strive for a sound balance between further development of nanotechnology and the necessary research to identify potential hazards in order to develop a scientifically defensible database for the purpose of risk assessment. To be able to do this, a basic knowledge about mammalian and ecotoxicologic profiles of NPs is necessary, rather than attempting to assess NP risks based on some popular science fiction literature. Most important, sufficient resources should be allocated by governmental agencies and industries to be able to perform a scientifically based risk assessment and then establish justifiable procedures for risk management. The data needed for this risk assessment should be determined a priori so that limited resources can be used efficiently to develop useful and well-planned studies.

At this point, governmental regulation is not possible, given the lack of needed information on which to base such regulations. However, academia, industry, and regulatory governmental agencies should seriously consider the view that NPs have new and unique biologic properties and that the potential risks of NPs are not the same as those of the bulk material of the same chemistry. Assigning a unique identifier to nanosized materials would indicate that the toxicology profile of the material in question may not be the same as the bulk material. Toxicologic tests and the resulting database would provide information for material safety data sheets for NPs as well as a basis for potential NP risk assessments and risk management. Obviously, this approach may not be appropriate for all NPs, for example, when embedded in a matrix, and the feasibility of this proposed strategy needs to be thoroughly discussed and considered. For discussing this, and for developing and deciding upon a reasonable battery of tests for toxicologic profiling, it would be very useful to convene international multidisciplinary workshops of experts from industry, academia, and regulatory agencies (including material scientists, chemists, chemical engineers, toxicologists, physicians, regulators, statisticians, and others) to establish an NP classification scheme and testing guidelines. A multidisciplinary and multinational collaborative team approach is critical. Respective efforts have been initiated nationally by the American National Standards Institute (ANSI 2004) and internationally by the International Council on Nanotechnology (ICON 2004) as well as the International Organization for Standardization (Geneva, Switzerland).

**Figure 15:**
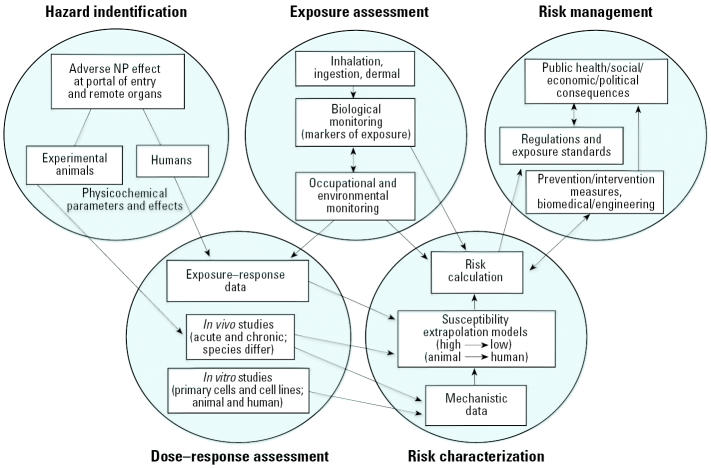
Risk assessment (NRC 1983) and risk management paradigm for NPs. Risk assessment requires answers to the following questions: Do NPs have adverse effects? What are the dose–response relationships? What are occupational/environmental levels in different media? What is the calculated risk? Once a risk is determined, a risk management decision can be established, including exposure standards and regulations and efforts for effective risk communication. Modified from Oberdörster (1994).

Because many regulatory agencies do not consider a nanotechnologically manufactured substance different from the conventional substance, the manufacture and use of nanotechnology products are currently not specifically regulated. Typically, nanosized substances are treated as variations of the technical material or existing formulation and thus do not require a separate registration. A main reason for producing a nanosize form of a registered substance, however, is that conversion of a substance to a nanoparticle imparts new properties to the substance (e.g., enhanced mechanical, electrical, optical, catalytic, biologic activity). Thus, as stated above, although the toxicology of the base material may be well defined, the toxicity of the nanosize form of the substance may be dramatically different from its parent form. As a result, new toxicology data on the nanosize form of a substance is likely to result in a different hazard assessment for the NPs. Figure 15 shows a risk assessment/risk management paradigm that points out different steps and data required for this process.

As described in the preceding sections, the difference in toxicologic profile of NPs compared with its parent form is due to not only its intrinsic chemical properties but also to a large degree to its differing kinetics *in vivo*. Although larger particles may not enter the CNS, the potential exists for inhaled NSPs to be translocated to the CNS via the axons of sensory neurons in the upper respiratory tract. Furthermore, although the toxicity per unit mass of a particular substance may vary depending on the nano versus larger form, it will be important to take into account not only new biologic activities but also potential new target organs and routes of exposure. To what degree does the nanoform of a substance have enhanced dermal penetration, or increased systemic uptake via the lung or GI tract? What determines how many nanoparticles that enter the systemic circulation will distribute throughout the body, reach the bone marrow, cross the blood–brain barrier, cross the placenta to affect the developing offspring, or sequester effectively in the liver? Do nanoparticles released into the environment affect species that are important in food chain dynamics? What are the long-term consequences of exposure to nanoparticles? Changes in toxicity profile and new target organs can be expected, and it will then be necessary to establish new risk assessments for nanoparticles in addition to the bulk material. Currently there exists a paucity of data to effectively address these questions, but it will be important to determine whether there exist common modes of action/behavior of NPs to establish baseline assumptions for use in risk assessments.

The use of nanotechnology products will likely increase dramatically over the next decade. In fact, nanomaterials are already being used in applications ranging from burn and wound dressings to dental-bonding agents to sunscreens and cosmetics to fuel cells, tires, optics, clothing, and electronics. Although currently there exists little public awareness of nanotechnology in everyday life (e.g., stain-free clothing), it would be prudent to examine and address environmental and human health concerns before the widespread adoption of nanotechnology. Both the societal benefits and potential risks of nanotechnology should be evaluated and clearly communicated to the general public and regulators. This type of open communication and risk/benefit evaluation will avoid the pitfalls encountered with genetically modified organisms recently experienced in the field of biotechnology. In that instance, the benefits of the emerging field of biotechnology were not communicated effectively before the introduction of the technology. As the public’s awareness of this new technology grew, regulators and producers of biotechnology failed to effectively acknowledge public concerns that genetically modified organisms could adversely affect ecosystem balance. As a result, the public support of genetically modified organisms, particularly in the European Union, is low. For nanomaterial producers, it will be important to demonstrate that what they may perceive as a new and potentially harmless form of a familiar material has, indeed, an acceptable risk profile. If such proactive steps are not taken, nanomaterials may be regarded as dangerous by the public and regulators, which could lead to inappropriate categorization and unnecessarily burdensome regulations. Such action (or inaction on the side of producers), in turn, could result in significant barriers to commercialization and the widespread acceptance of otherwise useful nanotechnology materials.

## Summary and Outlook

**Figure 16:**
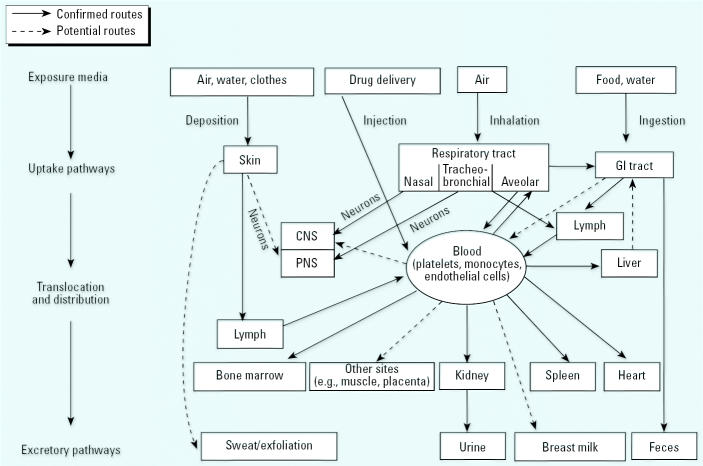
Biokinetics of NSPs. PNS, peripheral nervous system. Although many uptake and translocation routes have been demonstrated, others still are hypothetical and need to be investigated. Translocation rates are largely unknown, as are accumulation and retention in critical target sites and their underlying mechanisms. These, as well as potential adverse effects, largely depend on physicochemical characteristics of the surface and core of NSPs. Both qualitative and quantitative changes in NSP biokinetics in a diseased or compromised organism also need to be considered.

Research on ambient UFPs has laid the foundation for the emerging field of nanotoxicology, with the goal of studying the biokinetics and the potential of engineered nanomaterials (particles, tubes, shells, quantum dots, etc.) to cause adverse effects. Major differences between ambient UFPs and NPs are the polydisperse nature of the former versus the monodisperse size of the latter, and particle morphology, oftentimes a branched structure from combustion particles versus spherical form of NPs, although other shapes (tubes, wires, rings, planes) are also manufactured. In addition, combustion-derived volatile organic compounds and inorganic constituents (e.g., metals, nitrates, sulfates) of different solubilities on UFPs predict differences in the toxicologic profile between UFPs and NPs. However, as far as the insoluble particle is concerned, concepts of NSPs kinetics, including cell interactions, will most likely be the same for UFPs and NPs (Figure 16).

The introduction of nanostructured materials for biomedical and electronics applications opens tremendous opportunities for biomedical applications as therapeutic and diagnostic tools as well as in the fields of engineering, electronics, optics, consumer products, alternative energy, soil/water remediation, and others. However, very little is yet known about their potential to cause adverse effects or humoral immune responses once they are introduced into the organism—unintentionally or intentionally. Nanomedicine products will be well tested before introduction into the marketplace. However, for the manufacturers of most current nanotechnology products, regulations requiring nanomaterial-specific data on toxicity before introduction into the marketplace are an evolving area and presently under discussion (Bergeson and Auerbach 2004; Foresight and Governance Project 2003). During a product’s life cycle (manufacture, use, disposal), it is probable that nanomaterials will enter the environment, and currently there is no unified plan to examine ecotoxicologic effects of NPs. In addition, the stability of coatings and covalent surface modifications need to be determined both in ecologic settings and *in vivo*. [Supplemental Material available online (http://ehp.niehs.nih.gov/members/2005/7339/supplemental.pdf).]

Results of older biokinetic studies and some new toxicology studies with NSPs (mostly ambient UFPs) can be viewed as the basis for the expanding field of nanotoxicology. These studies showed that the greater surface area per mass renders NSPs more active biologically than larger-sized particles of the same chemistry, and that particle surface area and number appear to be better predictors for NSPs-induced inflammatory and oxidative stress responses. The following emerging concepts of nanotoxicology can be identified from these studies:

The biokinetics of NSPs are different from larger particles. When inhaled, they are efficiently deposited in all regions of the respiratory tract; they evade specific defense mechanisms; and they can translocate out of the respiratory tract via different pathways and mechanisms (endocytosis and transcytosis). When in contact with skin, there is evidence of penetration to the dermis followed by translocation via lymph to regional lymph nodes. A possible uptake into sensory nerves needs to be investigated. When ingested, systemic uptake via lymph into the organism can occur, but most are excreted via feces. When in blood circulation, they can distribute throughout the organism, and they are taken up into liver, spleen, bone marrow, heart, and other organs. In general, translocation rates are largely unknown; they are probably very low but are likely to change in a compromised/diseased state.

The biologic activity and biokinetics are dependent on many parameters: size, shape, chemistry, crystallinity, surface properties (area, porosity, charge, surface modifications, weathering of coating), agglomeration state, biopersistence, and dose. These parameters are likely to modify responses and cell interactions, such as a greater inflammatory potential than larger particles per given mass, translocation across epithelia from portal of entry to other organs, translocation along axons and dendrites of neurons, induction of oxidative stress, pro-oxidant and antioxidant activity of NSPs in environmentally relevant species, binding to proteins and receptors, and localization in mitochondria.

The principles of cellular and organismal interactions discussed in this article should be applicable for both ambient UFPs and NPs, even if the latter are coated with a bio-compatible material. Knowledge about the bio-persistence of this coating is as essential as is knowledge about the bioavailability of the core material that could have intrinsic toxic properties, for example, semiconductor metal compounds in sub-10-nm quantum dots consisting of cadmium and lead compounds. The very small size of these materials makes them available to the same translocation processes described here for polydisperse NSPs, possibly even in a more efficient way because of their uniform size. When studying biologic/toxicologic effects, new processes of interactions with subcellular structures (e.g., microtubuli, mitochondria) will likely be discovered. The diversity of engineered nanomaterials and of the potential effects represents major challenges and research needs for nanotoxicology, including also the need for assessing human exposure during manufacture and use. The goal to exploit positive aspects of engineered nanomaterials and avoid potential toxic effects can best be achieved through a multidisciplinary team effort involving researchers in toxicology, materials science, medicine, molecular biology, bioinformatics, and their subspecialties.

## Correction

The authors found additional information on GI tract uptake of NSPs that was not in the original manuscript published online. This information has been included here in “Exposure via GI Tract and Skin.”

## Figures and Tables

**Figure 1:**
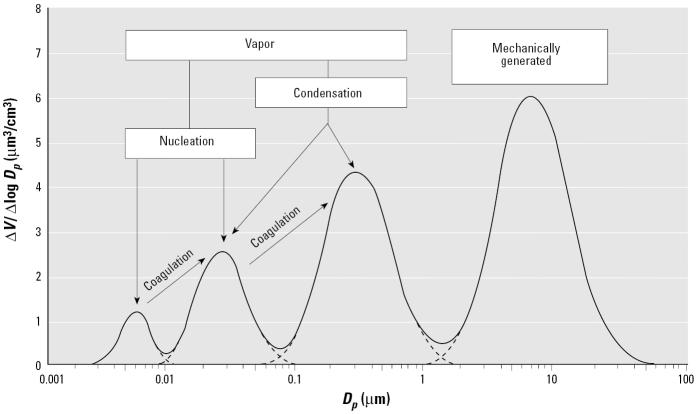
Idealized size distribution of traffic-related particulate matter (U.S. EPA 2004). *D_p_*, particle diameter. The four polydisperse modes of traffic-related ambient particulate matter span approximately four orders of magnitude from < 1 nm to > 10 μm. Nucleation- and Aitken-mode particles are defined as UFPs (< approximately 100 nm). Source-dependent chemical composition is not well controlled and varies considerably. In contrast, NPs (1–100 nm) have well-controlled chemistry and are generally monodispersed.

**Figure 2:**
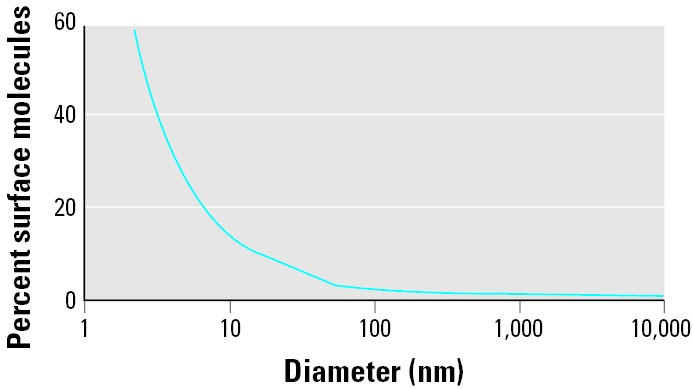
Surface molecules as a function of particle size. Surface molecules increase exponentially when particle size decreases < 100 nm, reflecting the importance of surface area for increased chemical and biologic activity of NSPs. The increased biologic activity can be positive and desirable (e.g., antioxidant activity, carrier capacity for therapeutics, penetration of cellular barriers), negative and undesirable (e.g., toxicity, induction of oxidative stress or of cellular dysfunction), or a mix of both. Figure courtesy of H. Fissan (personal communication).

**Table 1:** UFPs/NPs (< 100 nm), natural and anthropogenic sources.

	Anthropogenic	
Natural	Unintentional	Intentional (NPs)
Gas-to-particle conversions	Internal combustion engines	Controlled size and shape, designed for functionality
Forest fires	Power plants	
Volcanoes (hot lava)	Incinerators	Metals, semiconductors, metal oxides, carbon, polymers
Viruses	Jet engines	
Biogenic magnetite: magnetotactic	Metal fumes (smelting, welding, etc.)	Nanospheres, -wires, -needles, -tubes, -shells, -rings, -platelets
bacteria protoctists, mollusks, arthropods, fish, birds	Polymer fumes	
human brain, meteorite (?)	Other fumes	Untreated, coated (nanotechnology applied to many products: cosmetics, medical, fabrics, electronics, optics, displays, etc.)
Ferritin (12.5 nm)	Heated surfaces	
Microparticles (< 100 nm; activated cells)	Frying, broiling, grilling Electric motors	

**Table 2:** Particle number and particle surface area per 10 μg/m^3^ airborne particles.

Particle diameter (μm)	Particle no. (cm^–3^)	Particle surface area (μm^2^/cm^3^)
5	153,000,000	12,000
20	2,400,000	3,016
250	1,200	240
5,000	0.15	12

**Table 3:** Clearance mechanisms for inhaled solid particles in the respiratory tract.

Physical clearance processes (translocation)
Mucociliary movement (nasal, tracheobronchial)
Macrophage phagocytosis (tracheobronchial, alveolar)
Epithelial endocytosis (nasal, tracheobronchial, alveolar)
Interstitial translocation (tracheobronchial, alveolar)
Lymphatic drainage (tracheobronchial)
Blood circulation (tracheobronchial, alveolar)
Sensory neurons (nasal, tracheobronchial)
Chemical clearance processes*^a^*
Dissolution
Leaching
Protein binding

a Nasal, tracheobronchial, and alveolar regions.

**Table 4:** Particle size and surface chemistry-related alveolar–capillary translocation.

Particle size (nm)	Type	Translocation	Localization/effect	Reference
5–20	Gold, albumin coated	Yes	Via caveolae	Mehta et al. 2004
8	Gold, albumin coated	Yes	Via “vesicles”	König et al. 1993
8	Gold, albumin coated	Yes	Via caveolae	Heckel et al. 2004
18	Iridium	Yes*^a^*	Extrapulmonary organs	Kreyling et al. 2002
30	Gold	Yes	Platelet?	Berry et al. 1977
35	Carbon	Yes	Liver	Oberdörster et al. 2002
60	Polystyrene*^b^*	Yes	Thrombus, early	Nemmar et al. 2002b
				Silva et al., in press
60	Polystyrene	?	No thrombus	Nemmar et al. 2002b
80	Iridium	Yes*^a^*	Extrapulmonary organs	Kreyling et al. 2002
240	Polystyrene, lecithin	Yes	Monocytes	Kato et al. 2003
240	Polystyrene, uncoated	No		Kato et al. 2003
400	Polystyrene	No	Thrombus, late	Nemmar et al. 2003

?, unknown.

a Minimal.

b Indirect evidence.

**Table 5:** Translocation of NSPs in the blood circulation to bone marrow in mice.

Particle size	Type	Finding	Reference
~10 nm	PEG quantum dots	Fast appearance of quantum dots in liver, spleen, lymph nodes, and bone marrow (mouse)	Ballou et al. 2004
< 220 nm	Metallo-fullerene	Highest accumulation in bone marrow after liver; continued increase in bone marrow but decrease in liver (mouse)	Cagle et al. 1999
90–250 nm	HSA-coated polylactic acid nanoparticles	Significant accumulation in bone marrow, second to liver (rat)	Bazile et al. 1992
240 nm	Polystyrene (nonbiodegradable) polylisohexylcyonacrylate (biodegradable)	Rapid passage through endothelium in bone marrow, uptake by phagocytizing cells in tissue (mouse)	Gibaud et al. 1996, 1998, 1994

HSA, human serum albumin.

**Table 6:** Studies of neuronal translocation of UFPs from respiratory tract.

Reference	Study
Bodian and Howe 1941	Olfactory axonal transport of polio virus (30 nm) after intranasal instillation in chimpanzee; transport velocity, 2.4 mm/hr
de Lorenzo 1970	Olfactory axonal transport of 50 nm silver-coated gold after intranasal instillation in squirrel monkey; transport velocity, 2.5 mm/hr
Hunter and Dey 1998	Retrograde tracing of trigeminal neurons from nasal epithelium with microspheres
Hunter and Undem 1999	Rhodamine-labeled microspheres (20–200 nm) translocation via sensory nerves of TB region to ganglion nodosum in hamster after intratracheal instillation
Oberdörster et al. 2004	^13^C particles (CMD ~ 36 nm) in olfactory bulb after whole-body inhalation exposure in rats

TB, tracheobronchial.

**Table 7:** Rat versus human nasal and olfactory parameters.

Measure	Rat	Human
Breathing mode	Obligatory nose	Nasal/oronasal
Area of nasal mucosa	~16 cm^3^	~105 cm^2^
Area of olfactory mucosa (% total mucosa)	~8 cm^3^ (50)	~5.25 cm^2^ (5)
Percent nasal airflow going to olfactory mucosa	~15	~10
Weight of olfactory bulb	~85 ng	~168 ng

Based on Keyhani et al. (1997), Kimbell et al. (1997), and Turetsky et al. (2003).
