# Errata

**Published:** 2010-09

Oberdörster et al. have reported errors in their article “Nanotoxicology: An Emerging Discipline Evolving from Studies of Ultrafine Particles” ( Oberdörster et al. 2005. Environ Health Perspect 113:823–839):

- In Table 2 (p. 825), the units for particle diameter (heading of the first column) should be “nm” instead of “μm.”
- In Figure 8 (p. 829), the labels on the *y*-axis of each graph should be “Regional deposition fraction” (as stated correctly in the figure legend) instead of “Regional deposition (%).”
- In Table 4 (p. 832) under “Localization/effect,” the effect of 400 nm polystyrene (last row) should be “No thrombus” instead of “Thrombus, late”; this is described in more detail in the Supplemental Material (http://ehp.niehs.nih.gov/members/2005/7339/supplemental.pdf).

The authors apologize for the errors.

